# Dietary Intakes of EPA and DHA Omega-3 Fatty Acids among US Childbearing-Age and Pregnant Women: An Analysis of NHANES 2001–2014

**DOI:** 10.3390/nu10040416

**Published:** 2018-03-28

**Authors:** Zhiying Zhang, Victor L. Fulgoni, Penny M. Kris-Etherton, Susan Hazels Mitmesser

**Affiliations:** 1 Nutrition and Scientific Affairs, The Nature’s Bounty Co., Ronkonkoma, NY 11779, USA; susanhazels@hotmail.com; 2 Nutrition Impact LLC, Battle Creek, MI 49014, USA; vic3rd@aol.com; 3 Department of Nutritional Sciences, Pennsylvania State University, 110 Chandlee Laboratory, University Park, PA 16802, USA; pmk3@psu.edu

**Keywords:** seafood, EPA and DHA, dietary intake, childbearing-age and pregnant women, NHANES 2001–2014

## Abstract

Background: The 2015–2020 Dietary Guidelines for Americans (DGA) recommend that the general population should consume about 8 ounces (oz.) per week of a variety of seafood, providing approximately 250 mg per day of eicosapentaenoic acid (EPA) and docosahexaenoic acid (DHA), and that pregnant and lactating women should consume 8–12 oz. per week of seafood. Methods: We determined the usual intakes, percentage not meeting recommendations, and trends in EPA and DHA intakes among childbearing-age and pregnant women (15–44 years of age) using the NHANES cycles 2001–2002 through 2013–2014. Results: For the childbearing-age women, the mean usual intake of seafood was 0.44 ± 0.02 oz. equivalent per day and 100% of the population was below the DGA recommendation. Mean usual intakes of EPA, DHA, and combined EPA and DHA from foods and dietary supplements combined were 26.8 ± 1.4, 62.2 ± 1.9, and 88.1 ± 3.0 mg per day, respectively. Over 95% of the sample did not meet the daily intakes of 250 mg EPA and DHA. Similar results were observed for pregnant women. After controlling for covariates, there were slight but significant increases in EPA and DHA intakes from foods and dietary supplements over the 14-year span among childbearing-age (*p* = 0.005) and pregnant women (*p* = 0.002). Conclusions: It was estimated that a majority of U.S. childbearing-age and pregnant women consumed significantly lower amounts of seafood than what the DGA recommends, which subsequently leads to low intakes of EPA and DHA; in addition, dietary supplement use has not eliminated the nutrient shortfall.

## 1. Introduction

Eicosapentaenoic acid (EPA) and docosahexaenoic acid (DHA) are two important long-chain omega-3 fatty acids that contribute many beneficial health effects including cardiovascular health [1,2], brain [3,4], eye [5,6], and joint health [7,8], as well as fetal and infant development and maternal health [9,10]. Maternal plasma DHA levels were observed to be significantly increased during early pregnancy prior to neural tube closure, suggesting its contribution to embryo development [11]. DHA also rapidly accumulates in the human brain in the third trimester in the utero and during the first year after birth [12,13,14]; animal studies indicate that maternal omega-3 deficiency results in learning and behavior deficits of their offspring [15,16], which may be due to the predominant involvement of DHA in gene expression, synaptic plasticity, and neurogenesis [17,18,19,20]. EPA is not found in a large amount in the brain; however, recent animal studies reveal that the rate of EPA and DHA uptake into the brain is similar, but EPA is quickly metabolized by the brain through multiple pathways including energy production and elongation/desaturation to docosapentaenoic acid, the precursor of DHA [21,22]. EPA and DHA are not considered essential fatty acids as they can be derived from the desaturation and elongation of their parent fatty acid, alpha-linolenic acid (ALA); however, the conversion rate is low [23,24,25].

Unlike ALA, Dietary Reference Intakes (DRIs) have not been established for EPA and DHA. The National Academy of Medicine (formerly the Institute of Medicine, IOM) established an adequate intake (AI) for ALA and recommended that EPA and DHA contribute 10 percent of the total omega-3 fatty acid intake, providing approximately 160 mg per day [26]. Higher EPA and DHA intakes have been recommended by authoritative and expert scientific organizations worldwide (Table 1). For instance, the 2015–2020 Dietary Guidelines for Americans (DGA, 2015–2020) recommend that the general population consume 8 oz. of a variety of seafood per week (providing approximately 250 mg EPA and DHA per day), and up to 12 oz. per week for pregnant and lactating women in order to obtain nutrients, specifically DHA, for improved infant health [27]. In 2017, the FDA and the U.S. Environmental Protection Agency (FDA-EPA) released their advice on fish and shellfish consumption. Similar to the DGA’s guidance, the two agencies encourage young children and childbearing-age women, including those pregnant and breastfeeding, to eat more fish that is lower in mercury for important developmental and health benefits. Specifically, these populations should eat two to three servings of fish per week from the “Best Choices” list or one serving per week of fish from the “Good Choices” list [28]. Based on available evidence, Americans consume less fish/seafood than recommended, and the amount of EPA and DHA (Table 2) varies widely based on the type of seafood consumed [29]. According to the National Oceanic and Atmospheric Administration (NOAA), the top 10 seafoods consumed by Americans were shrimp, canned tuna, salmon, tilapia, Pollock, catfish, crab, cod, pangasius (certain types of freshwater catfish), and clams. Approximately 15.8 pounds of total fish and shellfish per person per year (~3.5 oz. per week on a per capita basis) is consumed, which is less than one-half of what the 2015–2020 DGA recommends [29].

Seafood consumption, including EPA and DHA, has been reported in many studies. Papanikolaou [39] studied adult men and non-pregnant/lactating women in NHANES 2003–2008 and reported that the mean usual intake of total fish was 0.61 oz. per day and EPA and DHA intake from food alone was 23 mg and 63 mg per day, respectively. EPA and DHA consumption from food and dietary supplements was 41 mg and 72 mg per day, respectively. Considering the potential conversion from ALA to EPA (5% conversion rate) and from stearidonic acid to EPA (SDA; 33% conversion rate), Richter [40] studied 24,621 subjects including children and adults in NHANES 2003–2008 and reported that the mean intake of EPA, DHA, and EPA equivalent from foods was 170 mg per day. In a study of 1260 pregnant and 5848 non-pregnant women 16–49 years of age from NHANES 1999–2006 [41], women consumed less than two servings of seafood a week, regardless of pregnancy status. The mean EPA and DHA intakes among U.S. childbearing women, including pregnant women (NHANES 2003–2012), were reported to be lower than the recommendations [42]; however, only one single day of dietary intake for each subject was included in the analysis. In the present study, we used the National Cancer Institute (NCI) method [43], which allows for determining the mean usual intake (estimate of long-term intake) and a less biased estimate of the percentage of the population who do not meet the dietary recommendation for seafood consumption and intakes of EPA and DHA. In addition, we quantified trends in EPA and DHA intakes over a 14-year span (NHANES 2001–2014).

## 2. Methods

### 2.1. The Continuous NHANES

The continuous NHANES is a large, nationally-representative, cross-sectional survey administered by the National Center for Health Statistics that is designed to evaluate the health and nutrition status among the U.S. population. Details of survey design, as well as data/sample collection, are available elsewhere [44]. In brief, data and samples are collected continuously and are released in two-year increments beginning in 1999. Since NHANES 1999–2000 nutrition data was a transition year for the USDA, the present study only combined data from NHANES 2001–2002 through 2013–2014 to increase statistical power and to assess trends in intake.

### 2.2. Subjects

All women of childbearing age (15–44 years of age) who provided complete demographic (age, race, and poverty-to-income ratio) information, questionnaire information (smoking status), and reliable 24-h dietary recall meeting the minimum criteria established by USDA Food and Nutrition Surveys staff, were included in the analysis. Pregnancy status was determined by the variable Pregnancy Status at Exam (RIDEXPREG) of Demographics data, as well as the variable Pregnancy Test (URXPREG) of Laboratory data.

### 2.3. Statistical Analysis

SAS 9.2 and SUDDAN 11 were used for the data analysis; survey parameters including strata, primary sampling units, and recommended sample weights, were used in all analyses. Mean usual intake of seafood, EPA, and DHA from all foods and other food was estimated using the National Cancer Institute method (NCI 2.1 MIXTRAN and DISTRIB macros), stratifying for population groups of interest. The two-part model was used given seafood/EPA/DHA are consumed episodically [43]. The first part of the model estimates the probability of consuming seafood/EPA/DHA and the second part provides consumption day amounts from 24-h recalls and estimates within subject variation on consumption days. Covariates in the models included age, weekend (Friday–Sunday)/weekday (Monday–Thursday) of recall, recall day sequence (day 1 or day 2), and EPA/DHA dietary supplement use (yes/no). Mean usual DHA and EPA intake from supplements was determined using the amount and frequency of consumption reported from a NHANES supplement intake questionnaire, and intake from food plus supplements was calculated as the sum of supplement intake and daily individual intake from food. The percentage of the sample population consuming less than 8 oz. per week (1.14 oz. per day) of seafood and 250 mg per day DHA plus EPA was generated with the NCI program. SUDAAN PROC DESCRIPT was used for counts and estimated percentages for various demographic groups. SUDAAN PROC REGRESS was used to evaluate EPA and DHA intakes across demographic groups; COND_EFF statements with PROC DESCRIPT specify contrasts that were used for the comparisons across the groups. SUDAAN PROC REGRESS was also used to determine if any changes in EPA and DHA intakes occurred over the 14-year span. Initial analyses assessed both linear and quadratic terms for time, but since quadratic terms were not significant, only the linear component analyses are presented. To help adjust for potential changes in population characteristics over time, covariates in analyses of intake over time included age (15–30 years of age and 31–44 years of age), race (non-Hispanic White, non-Hispanic Black, and Mexican Americans), socioeconomic status (poverty income ratio, PIR < 1.35, 1.35–1.85, and >1.85), and smoking status (yes and no). Statistical significance was set at *p* < 0.05.

## 3. Results

### 3.1. Demographic Characteristics

A total of 11,465 childbearing-age women were included in the present study (Table 3); 53.8% of the population was 15–30 years of age, while 46.2% was 31–44 years of age; 62.0% of the population was non-Hispanic White, 14% was non-Hispanic Black, and 11.1% was Mexican Americans; 25.5% of the population had education below high school or equivalent degree (lower education), 23.3% had a college graduate and above degree (higher education), and 51.3% had education in between (mid-level education); 31.6% of the population had a poverty income ratio (PIR) of less than 1.35 (lower income), 57.9% had PIR > 1.85 (higher income), and 10.5% had PIR between 1.35 and 1.85; 18.5% of the population were smokers. Of the 11,465 childbearing-age women, 1180 were pregnant women; 67.4% were 15–30 years of age and 93.7% were non-smokers; the adjusted proportions of other variables were similar to those among the childbearing-age women (Table 3).

### 3.2. Mean Usual Intakes of Seafood

The mean usual intakes of seafood are presented in Table 4 (distribution of intake is available in Supplementary Materials Table S1). Among childbearing-age women, mean usual seafood intakes were 0.44 ± 0.02 oz. equivalent per day; 100% of the population was below 8 oz. eq. per week (approximately 1.14 oz. eq. per day), as recommended by the 2015–2020 DGA. Non-Hispanic Whites consumed significantly less seafood (0.36 ± 0.03 oz. eq.) compared to non-Hispanic Blacks (0.62 ± 0.05 oz. eq.; *p* < 0.0001) and Mexican Americans (0.46 ± 0.05 oz. eq.; *p* = 0.024). Women with a lower education level had a significantly lower seafood consumption (0.36 ± 0.03 oz. eq.) compared to their mid-level and higher education counterparts (0.45 ± 0.03 oz. eq.; *p* = 0.034 and 0.53 ± 0.05 oz. eq.; *p* = 0.004, respectively). Furthermore, non-smokers consumed more seafood than smokers (0.47 ± 0.02 vs. 0.33 ± 0.05 oz. eq.; *p* = 0.01). No significant differences were observed across the three poverty income ratio groups. The majority of seafood intake was from seafood low in omega-3 fatty acids as designated by USDA (0.33 ± 0.02 among childbearing-age women and 0.38 ± 0.06 oz. eq. among pregnant women, respectively; data not shown).

Among pregnant women, mean usual seafood intake was 0.44 ± 0.06 oz. eq. per day; 100% of the population was below the DGA recommendation. Non-Hispanic Black pregnant women consumed 0.88 ± 0.24 oz. eq. seafood, followed by Mexican American (0.59 ± 0.16 oz. eq.) and non-Hispanic White (0.23 ± 0.06 oz. eq.) pregnant women. Non-Hispanic White pregnant women consumed a significantly lower amount of seafood compared to their counterparts (*p* < 0.05 each). Pregnant women with higher education consumed less seafood (0.24 ± 0.07 oz. eq.) compared to their counterparts. Statistical differences were not observed among the age or the poverty income ratio groups.

### 3.3. Mean Usual Intakes of EPA and DHA from Foods and Dietary Supplements

For childbearing-age women, the mean usual intakes of EPA from foods and from combined foods and dietary supplements were 18.5 ± 0.7 and 26.8 ± 1.4 mg per day, respectively (Table 5; distribution of intake is available in Supplementary Materials Table S2). DHA intakes from foods and from combined foods and dietary supplements were 55.0 ± 1.8 and 62.2 ± 1.9 mg per day, respectively. EPA and DHA intakes from foods were 72.6 ± 2.3 and from combined foods and dietary supplements were 88.1 ± 3.0 mg per day. Compared to their counterparts, women who were older, non-Hispanic Black, with a higher education, a higher poverty income ratio, and who were non-smokers, consistently consumed more EPA and DHA from foods and from combined foods and dietary supplements. Over 95% (95.87%) of the population did not meet the recommended 250 mg EPA and DHA per day.

For pregnant women, the mean usual intakes of EPA from foods and from combined foods and dietary supplements were 20.0 ± 2.0 and 23.0 ± 2.1 mg per day, respectively (Table 6); DHA intakes from foods and from combined foods and dietary supplements were 60.3 ± 5.0 and 76.7 ± 6.4 mg per day, respectively; EPA and DHA intakes from foods were 78.7 ± 6.8 and from combined foods with dietary supplements were 97.7 ± 8.0 mg per day. Similar to the childbearing-age women, older pregnant women also consumed more EPA and DHA; however, the differences across all other demographic groups were not significant. Approximately 95% (94.48%) of the population did not meet the recommended 250 mg EPA and DHA per day.

### 3.4. Trends in the Mean Intakes of Combined EPA and DHA from Foods and Dietary Supplements

Among childbearing-age women, the intakes of combined EPA and DHA were 94.93 ± 5.95 mg per day for NHANES 2001–2004 (*n* = 2229) and 94.48 ± 5.31 mg per day for NHANES 2011–2014 (*n* = 2188), respectively (data not shown). For pregnant women, the intakes were 85.87 ± 10.92 mg per day for NHANES 2001–2004 (*n* = 487) and 117.33 ± 19.83 mg per day for NHANES 2011–2014 (*n* = 111), respectively (data not shown). After controlling for age, socioeconomic status (PIR), smoking status, and race, EPA and DHA intakes from both foods and dietary supplements among childbearing-age women significantly increased over the 14-year span, with a 3.5 mg increase per each 2-year cycle (*p* = 0.005; Table 7). The increase among pregnant women over the 14-year span was also significant, with a 7.6 mg increase per each 2-year cycle (*p* = 0.002).

## 4. Discussion

The present study demonstrates that childbearing-age women, including pregnant women, only consumed an average of 0.44 oz. eq. of seafood per day (approximately 3.08 oz. eq. per week), which is much less than the DGA (2015–2020) [27] and FDA-EPA recommendation for fish consumption advice [28], and the recommendation that pregnant/lactating women consume at least 8 oz. and up to 12 oz. of a variety of seafood per week. Although variable statistical significances were observed across the demographic groups, the results clearly show consistently low intakes of fish given that the entire population (100%) did not meet the recommendation. The mean usual intakes of EPA and DHA from foods were 72.6 ± 2.3 mg per day among childbearing-age women and 78.7 ± 6.8 mg per day among pregnant women; dietary supplements helped increase the nutrient intakes. However, the total intake of EPA and DHA remained low regardless of the pregnancy status, with about 95% of the population not meeting the recommended 250 mg per day; the total intakes of EPA and DHA from foods and/or dietary supplements were also lower than the recommendations world-wide (Table 1).

Many studies demonstrate the benefits of long-chain omega-3 fatty acids on maternal health. EPA and DHA supplementation contributes to prolonged gestational length, reducing the risk of preterm birth [9,45,46], although some evidence suggests contradictory results (19–21).

Perinatal depression is a devastating mental disorder that impacts not only childbearing-age women, but also their children and families. According to a nationally representative survey, 1 in 10 children have a mother who is depressed [47]. Observational studies demonstrate an inverse association between circulating EPA and DHA levels and depressive symptoms among pregnant women [48,49]. However, more clinical trials are required to ascertain the benefits of EPA and DHA intakes on perinatal depression improvement as the results are mixed [50,51]. For instance, 800 mg per day DHA supplementation during pregnancy did not significantly ameliorate postpartum depressive symptoms measured by the Edinburgh Postnatal Depression Scale [46]; whereas 3400 mg per day EPA and DHA supplementation for eight weeks during pregnancy favorably improved depression measured by the Hamilton Rating Scale for Depression, the Edinburgh Postnatal Depression Scale, and Beck Depression Inventory [52].

A significant number of studies have reported that maternal DHA consumption contributes to infant health and development. DHA rapidly accumulates in the fetus’s brain during the third intrauterine trimester and the first year after birth [12,13,14]. Not all [53,54,55], but some observational and clinical trials, demonstrate an increased risk of lower language development and visual acuity among offspring if mothers were not supplemented with DHA during pregnancy [56,57]; DHA supplementation during pregnancy contributed to cognitive development [57,58,59] and a higher fetal heart rate variability, an important indicator of a responsive and flexible autonomic nervous system [60]. 

Maternal EPA and DHA intakes also contribute to a protective effect on the immunity for their offspring. Appropriate immune responses help to eliminate antigens; however, overactivation of an immune response contributes to various immune disorders such as asthma and atopy. Higher maternal omega-6 intake and higher omega-6 levels in breast milk are associated with a higher prevalence of asthma and atopy, whereas higher maternal omega-3 intake helps prevent these disorders [61,62,63,64]. Several clinical trials indicate that maternal fish oil supplementation during pregnancy can reduce circulating biomarkers [65,66,67] and the severity of allergic phenotype [68,69,70] among their offspring.

Due to the importance of these long-chain omega-3 fatty acids, EPA and DHA have been recommended worldwide for pregnant and lactating women. Some of these recommendations encourage seafood consumption, whereas others specify the amount of EPA and DHA intakes (250–450 mg per day) and 200–300 mg per day for DHA (Table 1).

Nevertheless, the nutrient shortfall can still be significant since intakes among childbearing-age women, including pregnant and lactating women, vary widely. In the U.S., no Daily Values have been established for EPA and DHA and the dietary recommendations are food-specific rather than nutrient-specific. Both the DGA and FDA-EPA final fish consumption recommendation emphasizes that pregnant and lactating women consume fish and shellfish to obtain EPA and DHA. However, none of these recommendations specify the amount of these nutrients. Since EPA and DHA quantity differs among seafood, the intakes of EPA and DHA may vary significantly due to preferred consumption. For instance, according to the NOAA [29], shrimp consumption among the general population in the U.S. was 25.9% of total seafood consumption, followed by canned tuna (14.4%) and salmon (11.6%). Preferences among pregnant and non-pregnant women were also evaluated, with shrimp being the most frequently reported seafood, followed by tuna [41]. Various types of fish and shellfish that are recommended by the FDA-EPA Fish Advisory [28] were selected and the quantities of EPA and DHA are presented in Table 2. Following the dietary recommendations (two to three servings per week), shrimp, the most popular seafood, provides 45.0–67.4 mg per day EPA and DHA and tuna (light), the second most consumed seafood, provides 42.3–109.1 mg per day EPA and DHA. Salmon, the third most consumed seafood, provides a much higher amount EPA and DHA (465–698 mg per day); however, the amount that people consumed of the higher EPA- and DHA-containing fish is much lower. The DGA recommends that pregnant or lactating women consume up to 12 oz. per week of seafood that is lower in methyl mercury; this limit may be due to the potential neurotoxicant impact [71,72]. Although studies indicated that the benefits from fish consumption may counter the adverse influence of methyl mercury [73,74], women were reported to reduce the consumption of fish during their pregnancy [75,76]. Furthermore, analytical studies demonstrated that cooking method may result in EPA and DHA loss [77,78]; thus, EPA and DHA consumption from cooked seafood may be less than presented in Table 2. Without nutrition education, how to select seafood to meet the recommendations can be a challenge.

The fetus and infants maximize the uptake of nutrients including EPA and DHA from their mothers to meet the requirement during development [12,79,80,81]. Many clinical studies have shown that maternal EPA and DHA intakes from dietary supplements increase these nutrients in the maternal circulation [82,83,84], breast milk, and infant circulation [82,85,86,87], depending on the amount of EPA and DHA consumption. These results indicate that dietary supplements may be an effective complement to foods to help improve both maternal and offspring nutrition status.

The present study found that the EPA and DHA intakes over a 14-year span increased only slightly (3.5–7.6 mg per 2-year period) and thus intakes did not significantly change over time among childbearing-age and pregnant women in the U.S. Because of their established health benefits, authoritative organizations have been encouraged to establish a Daily Value for EPA and DHA. On one hand, public health organizations and associations should take actions to educate the public on the importance of EPA and DHA and to encourage childbearing-age, pregnant, and lactating women to consume recommended amounts of EPA and DHA from fish/seafood, fortified foods, and dietary supplements. In addition, the Supplemental Nutrition Assistance Program (SNAP) and the Special Supplemental Nutrition Program for Women, Infants, and Children (WIC) should be modified to require EPA- and DHA-rich foods and dietary supplements for program participants.

There are a few strengths of our study. First, to our knowledge, this is the first study to evaluate mean usual intakes of EPA and DHA among childbearing-age including pregnant women using the most recent data from NHANES (2001–2014). Second, the study reported the percentage of the population who do not meet the DGA’s dietary recommendation. The present study also reports the trend of EPA and DHA intakes over a 14-year span after controlling for covariates such as age, race, socioeconomic status, and smoking status. All of these factors help to further our understanding of the maternal nutrition shortfall, encouraging the implementation of nutrition education programs to increase the intake of EPA and DHA.

Limitations of this study include the fact that the NHANES data are cross-sectional and as such cause and effect cannot be determined, the dietary data are self-reported, and the sample size of pregnant women is relatively small (given the small sample sizes relative to the total population, it is possible that some childbearing-age women consume recommended amounts, but from our data, the percentage seems very low). However, the objective of the present study was to determine EPA and DHA intakes nationwide, and NHANES is the nationally representative database.

## 5. Conclusions

In conclusion, the present study adds to the growing evidence that childbearing-age women, including those that are pregnant, in the U.S. are not meeting dietary recommendations for seafood consumption, and as a result, exhibit low intakes of EPA and DHA. The maternal EPA and DHA intake gap is still obvious and is expected to have a long-term impact on both maternal and infant health. Authoritative organizations are urged to take action and implement nutrition education programs that increase EPA and DHA intake for the population, especially childbearing-age women, including those that are pregnant.

## Figures and Tables

**Table 1:** EPA and DHA worldwide recommendations for pregnant and lactating women.

Source	Note
Food and Agriculture Organization of the United Nations (FAO; 2010) [30]	300 mg per day EPA + DHA, of which 200 mg per day DHA
World Association of Perinatal Medicine (WAPM, 2008) [31]	200 mg per day DHA
Koletzko et al., Consensus recommendation on behalf of the European Commission research projects Perinatal Lipid Metabolism (PeriLip) and International Society for the Study of Fatty Acids and Lipids (ISSFAL) 2007 [32]	200 mg per day DHA; aiming to consume 1–2 portions of sea fish per week, including oily fish
European Food Safety Authority (EFSA), 2010 [33]	An additional 100–200 mg per day DHA beyond 250 mg per day EPA + DHA
Simopoulos et al., 1999; Workshop sponsored by NIH and ISSFAL [34]	300 mg per day DHA
Analysis of the balancing of benefits and risks of seafood consumption. In: Nesheim MC Yaktine AL, eds. Seafood choices: balancing benefits and risks. Washington, DC:, National Academies Press, 2007 [35]	Two 3 oz. (cooked) servings of higher EPA- and DHA-containing seafood per week
Coletta, et al., 2010; American College of Obstetricians and Gynecologists (ACOG) adopted FDA advise for pregnant women (2010) as well as Koletzko (2007) recommendations [36]	340 g (two 6 oz. servings) seafood per week, providing approximately 200 mg per day DHA
March of Dimes (U.S. National Foundation; 2009) [37]	200 mg per day DHA
American Academy of Pediatrics (AAP) Policy Statement—Breastfeeding and the use of human milk, 2012 [38]	200–300 mg per day DHA
Dietary Guidelines for Americans (DGA), 2015–2020 [27]	8 oz. per week of a variety of seafood (approximately 250 mg per day of EPA and DHA)
FDA-EPA final fish consumption advice, 2017 [28]	2–3 servings (approximately 8–12 oz.) of fish from the “Best Choices” or 1 serving of fish from the “Good Choices” (approximately 4 oz.)

**Table 2:** EPA and DHA quantities in selected seafood and amount provided from seafood recommendations ^1^.

**Selected Seafood from Best Choices** [28] **(in Descending Order by Amount Consumed in the US** [29]**)**	**USDA Code**	**2–3 Servings Per Week Recommended by FDA-EPA Final Advisory**		
		**EPA per Day (mg)**	**DHA per Day (mg)**	**EPA + DHA per Day (mg)**
Shrimp, Mixed species	15149	22.1–33.1	22.9–34.3	45.0–67.4
Tuna, light, canned in water	15121	9.1–13.7	63.6–95.4	72.8–109.1
Tuna, light, canned in oil	15119	9.1–13.7	33.1–49.7	42.3–63.4
Salmon, Atlantic	15076	104.0–156.0	361.1–541.7	465.1–697.7
Tilapia	15261	1.6–2.4	27.8–41.8	29.5–44.2
Pollock, Atlantic	15065	22.9–34.3	113.1–169.7	136.0–204.0
Catfish	15010	42.3–63.4	141.7–212.6	184.0–276.0
Crab, Blue	15139	55.2–82.9	48.8–73.1	104.0–156.0
Cod	15015	56.4–84.6	105.5–158.3	161.9–242.9
Clams, mixed species	15157	14.1–21.1	17.1–25.7	31.2–46.9
**Selected seafood from Good choices** [28] **(alphabetical ranking)**	**USDA code**	**1 serving per week recommended by FDA-EPA final advisory**		
		**EPA per day (mg)**	**DHA per day (mg)**	**EPA + DHA per day (mg)**
Bluefish	15005	81.5	168.0	249.5
Carp	15008	77.0	37.0	113.9
Grouper	15031	8.8	71.2	80.0
Spanish mackerel	15051	106.7	327.6	434.3
Tuna, yellowfin	15127	3.8	28.6	32.4

^1^ EPA and DHA quantities from selected seafood of “Best Choices” and “Good Choices” that the FDA-EPA advisory recommends, where people should consume two to three servings per week seafood from the “Best Choices” or one serving per week seafood from the “Good Choices”. In the “Best Choices” category, the order is presented according to the NOAA American’s most consumed seafood ranking. The selected seafood in the “Good Choices” category is not America’s most consumed according to the NOAA; therefore, the order is presented by alphabetical ranking.

**Table 3:** Demographic characteristics of subjects.

	Childbearing-Age		Pregnant	
Variable	*n* = 11,465	Weighted-Estimate Adjusted % (S.E)	*n* = 1180	Weighted-Estimate Adjusted % (S.E.)
Age (years)				
15–30 *	7121	53.8 (0.9)	864	67.4 (2.6)
31–44	4344	46.2 (0.9)	316	32.6 (2.6)
Race				
Non-Hispanic White	4370	62.0 (1.5)	494	52.7 (2.9)
Non-Hispanic Black	2694	14.0 (0.9)	217	17.8 (2.0)
Mexican Americans	2569	11.1 (0.8)	319	15.6 (1.8)
Education				
Less than high school or equiv.	4324	25.5 (0.7)	367	21.5 (1.8)
High school/equiv. & college/AA	5238	51.3 (0.9)	558	49.4 (2.3)
College graduate & above	1896	23.2 (0.9)	255	29.1 (2.4)
Poverty income ratio				
<1.35	4371	31.6 (1.0)	437	33.1 (2.3)
1.35–1.85	1222	10.5 (0.4)	122	11.6 (1.6)
>1.85	5154	57.9 (1.1)	555	55.4 (2.9)
Smoking status				
Yes	1655	18.5 (0.7)	75	6.3 (1.1)
No	9546	81.5 (0.7)	1104	93.7 (1.1)

* For confidentiality reasons, the publicly available data only reports pregnancy for those 20–44 years of age.

**Table 4:** Mean usual daily intake of seafood among childbearing-age and pregnant women for a fully qualified sample 2001–2014.

	Childbearing-Age Women		Pregnant Women	
	Seafood Intake per Day (oz. eq.) ^1^	% Below Recommendation ^2^	Seafood Intake per Day (oz. eq.) ^1^	% of Sample Not Meeting Recommendation ^2^
All	0.44 ± 0.02	100%	0.44 ± 0.06	100%
Age (years)				
15–30	0.36 ± 0.02 ^a^	100%	0.39 ± 0.07	100%
31–44	0.54 ± 0.03 ^b^	100%	0.56 ± 0.10	100%
Race				
Non-Hispanic White	0.36 ± 0.03 ^a^	100%	0.23 ± 0.06 ^a^	100%
Non-Hispanic Black	0.62 ± 0.05 ^b^	100%	0.88 ± 0.24 ^b^	100%
Mexican Americans	0.46 ± 0.05 ^a^	100%	0.59 ± 0.16 ^b^	100%
Education				
Less than high school or equiv.	0.36 ± 0.03 ^a^	100%	0.56 ± 0.17 ^a,b^	100%
High school/equiv. & college/AA	0.45 ± 0.03 ^b^	100%	0.54 ± 0.08 ^b^	100%
College graduate & above	0.53 ± 0.05 ^b^	100%	0.24 ± 0.07 ^a^	100%
Poverty income ratio				
<1.35	0.40 ± 0.03	100%	0.48 ± 0.14	99.9%
1.35–1.85	0.42 ± 0.07	99.99%	0.50 ± 0.14	100%
>1.85	0.46 ± 0.03	100%	0.44 ± 0.08	100%
Smoking status				
Yes	0.33 ± 0.05 ^a^	100%	NA ^3^	NA ^3^
No	0.47 ± 0.02 ^b^	100%	0.46 ± 0.07	100%

^1^ Seafood intakes per day are presented as mean ± SEM. (ounce equivalent). ^a,b^ Means in a column of each variable with superscripts without a common letter differ, *p* < 0.05. ^2^ Percentage below recommendation refers to the percentage of childbearing-age and pregnant women who do not consume at least 8 oz. of seafood per week (or 1.14 oz. eq. per day) as recommended by Dietary Guidelines for Americans (2015–2020). ^3^ NCI usual intake programing requires at least one subject to have a positive intake on two or more 24-h recalls in order to converge. There were no subjects that met this requirement in the pregnant/smoking = yes sample population.

**Table 5:** Mean usual daily intake of EPA and DHA from foods alone, and from foods and dietary supplements among childbearing-age women for a fully qualified sample 2001–2014 ^1.^

	EPA Intake (mg)		DHA Intake (mg)		EPA + DHA Intake (mg)		% of Sample Not Meeting 250 mg/d ^2^
	Foods	Foods + Supplements	Foods	Foods + Supplements	Foods	Foods + Supplements	
All	18.5 ± 0.7	26.8 ± 1.4	55.0 ± 1.8	62.2 ± 1.9	72.6 ± 2.3	88.1 ± 3.0	95.87 (0.50)
Age (years)							
15–30	16.9 ± 0.6 ^a^	21.7 ± 1.2 ^a^	48.8 ± 1.7 ^a^	53.6 ± 1.9 ^a^	65.5 ± 2.2 ^a^	75.0 ± 2.8 ^a^	97.42 (0.42) ^b^
31–44	20.3 ± 0.9 ^b^	32.8 ± 2.3 ^b^	62.2 ± 2.6 ^b^	72.2 ± 2.9 ^b^	80.8 ± 3.2 ^b^	103.5 ± 4.7 ^b^	94.08 (0.73) ^a^
Race							
Non-Hispanic White	15.1 ± 0.9 ^a^	25.0 ± 1.8 ^a,b^	44.6 ± 2.4 ^a^	52.9 ± 2.5 ^a^	58.6 ± 3.0 ^a^	76.7 ± 3.9 ^a^	96.46 (0.46) ^b^
Non-Hispanic Black	26.7 ± 1.8 ^b^	29.5 ± 1.9 ^b^	68.4 ± 4.0 ^b^	71.4 ± 4.0 ^b^	102.8 ± 6.3 ^b^	108.2 ± 6.3 ^b^	93.52 (1.31) ^a^
Mexican Americans	18.2 ± 1.4 ^a^	21.2 ± 1.4 ^a^	62.2 ± 3.5 ^b^	65.5 ± 3.6 ^b^	80.2 ± 5.0 ^c^	87.0 ± 5.0 ^a^	97.35 (0.76) ^b^
Education							
Less than high school or equiv.	14.6 ± 0.9 ^a^	18.6 ± 2.3 ^a^	46.4 ± 2.5 ^a^	49.8 ± 2.9 ^a^	60.3 ± 3.2 ^a^	67.8 ± 4.8 ^a^	98.87 (0.45) ^c^
High school/equiv. & college/AA	17.9 ± 0.9 ^b^	24.8 ± 1.8 ^b^	54.4 ± 2.3 ^b^	59.9 ± 2.6 ^b^	71.6 ± 3.1 ^b^	84.3 ± 4.0 ^b^	96.46 (0.66) ^b^
College graduate & above	24.8 ± 2.0 ^c^	40.8 ± 2.8 ^c^	64.4 ± 4.7 ^c^	78.9 ± 4.7 ^c^	87.9 ± 6.2 ^c^	118.2 ± 6.8 ^c^	90.87 (1.18) ^a^
Poverty income ratio							
<1.35	16.0 ± 0.8 ^a^	21.0 ± 1.5 ^a^	49.3 ± 2.4 ^a^	53.4 ± 2.6 ^a^	64.4 ± 3.00 ^a^	73.6 ± 3.7 ^a^	98.01 (0.46) ^b^
1.35–1.85	16.0 ± 1.6 ^a^	24.1 ± 5.0 ^a,b^	50.3 ± 4.7 ^a,b^	56.7 ± 6.4 ^a,b^	64.0 ± 5.8 ^a^	78.5 ± 10.7 ^a,b^	95.85 (1.19) ^a,b^
>1.85	20.0 ± 1.1 ^b^	30.7 ± 1.9 ^b^	57.4 ± 2.9 ^b^	66.6 ± 3.0 ^b^	77.0 ± 3.8 ^b^	96.7 ± 4.6 ^b^	94.89 (0.76) ^a^
Smoking status							
Yes	13.1 ± 1.1 ^a^	19.5 ±2.9 ^a^	40.4 ± 2.9 ^a^	45.5 ± 3.7 ^a^	52.9 ± 3.8 ^a^	64.6 ± 6.1 ^a^	98.11 (0.61) ^b^
No	19.9 ± 0.8 ^b^	28.8 ± 1.5 ^b^	58.7 ± 2.1 ^b^	66.5 ± 2.1 ^b^	77.6 ± 2.8 ^b^	94.4 ± 3.3 ^b^	95.27 (0.63) ^a^

^1^ Usual daily intake of EPA and DHA is presented as mean ± SEM. ^a,b,c^ Means in a column of each variable with superscripts without a common letter differ, *p* < 0.05. ^2^ Percentage of population not meeting recommendation is presented as percent (S.E.). Percentage below recommendation refers to the percentage of childbearing-age and pregnant women who do not consume at least 250 mg EPA and DHA per day as recommended by Dietary Guidelines for Americans (2015–2020).

**Table 6:** Mean usual daily intake of EPA and DHA from foods alone, and from foods and dietary supplements among pregnant women for a fully qualified sample 2001–2014 ^1.^

	EPA Intake (mg)		DHA Intake (mg)		EPA + DHA Intake (mg)		% of Population Not Meeting Recommendation ^2^
	Foods	Foods + Supplements	Foods	Foods + Supplements	Foods	Foods + Supplements	
All	20.0 ± 2.0	23.0 ± 2.1	60.3 ± 5.0	76.7 ± 6.4	78.7 ± 6.8	97.7 ± 8.0	94.48 (1.48)
Age (years)							
20–30	18.8 ± 2.1	20.0 ± 2.1 ^a^	57.6 ± 5.0	66.7 ± 6.6 ^a^	75.3 ± 7.1	85.1 ± 8.3 ^a^	96.35 (1.26)
31–44	22.2 ± 2.8	29.5 ± 3.9 ^b^	65.1 ± 8.8	98.5 ± 12.5 ^b^	84.5 ± 10.2	123.3 ± 15.0 ^b^	90.41 (3.26)
Race							
Non-Hispanic White	12.6 ± 1.6 ^a^	16.0 ± 2.1 ^a^	38.9 ± 4.7 ^a^	58.4 ± 8.9	50.1 ± 5.5 ^a^	72.67 ± 9.6	95.7 (2.13)
Non-Hispanic Black	35.1 ± 8.9 ^b^	35.4 ± 8.8 ^b^	80.5 ± 14.3 ^b^	80.0 ± 14.2	115.4 ± 23.9 ^b^	116.7 ± 24.0	91.97 (6.11)
Mexican Americans	22.5 ± 5.2 ^a,b^	24.0 ± 5.2 ^a,b^	79.8 ± 14.3 ^b^	88.1 ± 14.9	99.6 ± 19.5 ^b^	109.3 ± 20.2	96.43 (2.04)
Education							
Less than high school or equiv.	24.5 ± 5.4	25.8 ± 5.3	73.7 ± 12.6 ^a,b^	83.9 ± 14.3	101.1 ± 20.5	112.6 ± 20.9	97.35 (1.86)
High school/equiv. & college/AA	20.6 ± 2.8	22.6 ± 2.8	61.2 ± 5.9 ^b^	72.2 ± 7.7	79.3 ± 8.4	92.3 ± 9.3	93.62 (2.14)
College graduate & above	16.4 ± 3.7	22.7 ± 4.3	48.8 ± 9.3 ^a^	80.3 ± 14.8	64.3 ± 15.0	101.5 ± 20.3	92.17 (3.24)
Poverty income ratio							
<1.35	20.6 ± 3.5	22.9 ± 3.6	60.9 ± 8.1	64.8 ± 8.2	76.8 ± 10.7	85.1 ± 10.4	96.17 (2.04)
1.35–1.85	18.9 ± 4.6	21.4 ± 4.8	59.3 ± 11.7	70.0 ± 13.9	76.6 ± 15.8	86.2 ± 19.3	94.45 (4.68)
>1.85	20.6 ± 2.7	24.6 ± 3.2	64.8 ± 8.5	90.3 ± 12.4	84.3 ± 10.4	112.8 ± 15.9	93.40 (2.61)
Smoking status							
Yes	12.9 ± 5.8	14.4 ± 5.9	53.8 ± 16.9	54.9 ± 16.9	64.3 ± 21.1	66.7 ± 20.9	99.14 (0.98) ^b^
No	20.5 ± 2.1	23.8 ± 2.3	60.6 ± 5.1	78.5 ± 6.8	79.0 ± 7.1	99.8 ± 8.6	93.89 (1.58) ^a^

^1^ Usual daily intake of EPA and DHA is presented as mean ± SEM. ^a,b^ Means in a column of each variable with superscripts without a common letter differ, *p* < 0.05. ^2^ Percentage of population not meeting recommendation is presented as percent (S.E.). Percentage below recommendation refers to the percentage of childbearing-age and pregnant women who do not consume at least 250 mg EPA and DHA per day as recommended by Dietary Guidelines for Americans (2015–2020).

**Table 7:** Change of EPA and DHA intakes (from foods and dietary supplements combined) over 14-year span ^1^.

	Childbearing-Age Women		Pregnant Women	
Variables	β (S.E.)	*p*-Value	β (S.E.)	*p*-Value
Cycle	3.5 (1.2)	0.005	7.6 (2.5)	0.002
Age (vs. 15–25 years of age)				
26–44 years of age	11.5 (3.3)	<0.001	−6.2 (10.9)	0.574
PIR (vs. <1.35)				
1.35–1.85	5.0 (8.2)	0.544	8.4 (9.0)	0.353
>1.85	6.0 (3.3)	0.066	20.7 (8.3)	0.014
Smoking (vs. No)				
Yes	−4.4 (5.3)	0.411	−5.4 (5.3)	0.309
Race (vs. Non-Hispanic White)				
Non-Hispanic Black	−12.0 (3.4)	0.001	−12.5 (5.4)	0.023
Mexican American	−11.0 (3.4)	0.002	1.4 (7.4)	0.847

^1^ Model examines the change of mean EPA and DHA intakes over 14 years (2001–2014). The regression model was adjusted for survey socioeconomic status (PIR), age, smoking status, and race, whereas education was not included in the final model due to collinearity with PIR.

## Supplementary Materials

The following are available online at http://www.mdpi.com/2072-6643/10/4/416/s1, Table S1: Distribution of seafood intake (ounce equivalent) in childbearing-age women and pregnant women, Table S2: Distribution of EPA and DHA intake (mg per day) from food alone and from food plus dietary supplements in childbearing-age women and pregnant women.
