# Supplementary material for: Dietary Intakes of EPA and DHA Omega-3 Fatty Acids among US Childbearing-Age and Pregnant Women: An Analysis of NHANES 2001–2014
Source: Nutrients. 2018 Mar 28;10(4):416. doi: 10.3390/nu10040416 (PMC5946201; doi:10.3390/nu10040416)
Supplement: Supplementary file 1 [file nutrients-10-00416-s001.pdf]

Supplemental Table 1. Distribution of seafood intake (ounce equivalent) in childbearing-age women and pregnant women.

	**Childbearing-age women**										
	**N**	**P10**	**SE**	**P25**	**SE**	**P50**	**SE**	**P75**	**SE**	**P90**	**SE**
**All**	10,285	0.08	0.02	0.16	0.03	0.32	0.03	0.59	0.03	0.98	0.09
**Age (years)**											
15-30	6,257	0.07	0.02	0.13	0.03	0.25	0.03	0.48	0.03	0.80	0.08
31-44	4,028	0.12	0.03	0.21	0.04	0.40	0.04	0.72	0.05	1.14	0.11
**Race**											
Non-Hispanic White	3,876	0.06	0.03	0.11	0.04	0.23	0.04	0.47	0.04	0.83	0.12
Non-Hispanic Black	2,477	0.14	0.04	0.26	0.06	0.49	0.07	0.83	0.07	1.23	0.13
Mexican Americans	2,250	0.15	0.07	0.24	0.07	0.39	0.06	0.60	0.07	0.85	0.14
**Education**											
Less than high school or equiv.	3,957	0.08	0.03	0.13	0.03	0.25	0.03	0.46	0.05	0.75	0.10
High school/equiv. & college/AA	4,680	0.07	0.03	0.15	0.04	0.30	0.05	0.59	0.05	1.01	0.12
College graduate & above	1,641	0.12	0.05	0.21	0.06	0.40	0.07	0.72	0.07	1.17	0.15
**Poverty income ratio**											
<1.35	3,934	0.11	0.05	0.19	0.06	0.32	0.05	0.52	0.04	0.77	0.12
1.35-1.85	1,100	0.01	0.02	0.05	0.04	0.15	0.07	0.46	0.10	1.11	0.22

>1.85	4,599	0.08	0.03	0.16	0.04	0.32	0.05	0.61	0.05	1.03	0.12
**Smoking status**											
Yes	1,580	0.07	0.06	0.13	0.07	0.25	0.07	0.44	0.07	0.71	0.16
No	8,442	0.10	0.03	0.18	0.03	0.35	0.04	0.64	0.04	1.02	0.10
	**Pregnant women**										
	**N**	**P10**	**SE**	**P25**	**SE**	**P50**	**SE**	**P75**	**SE**	**P90**	**SE**
**All**	1,180	0.06	0.05	0.12	0.07	0.28	0.08	0.61	0.09	1.07	0.18
**Age (years)**											
15-30	864	0.05	0.04	0.11	0.06	0.25	0.07	0.53	0.10	0.96	0.21
31-44	316	0.08	0.08	0.17	0.10	0.39	0.12	0.80	0.15	1.34	0.24
**Race**											
Non-Hispanic White	494	0.01	0.02	0.04	0.03	0.11	0.06	0.30	0.11	0.62	0.17
Non-Hispanic Black	217	0.52	0.29	0.66	0.25	0.85	0.26	1.07	0.33	1.30	0.54
Mexican Americans	319	0.36	0.15	0.43	0.13	0.53	0.14	0.70	0.24	0.96	0.46
**Education**											
Less than high school or equiv.	367	0.14	0.11	0.26	0.27	0.45	0.26	0.74	0.24	1.10	0.52
High school/equiv. & college/AA	558	0.11	0.08	0.20	0.11	0.39	0.12	0.72	0.14	1.17	0.24
College graduate & above	255	0.01	0.03	0.04	0.05	0.12	0.07	0.33	0.10	0.67	0.30

Data source: National Health and Nutrition Examination Survey 2001-2014; usual intake estimated using the National Cancer Institute method

**Poverty income ratio**										
<1.35	437	0.03	0.09	0.07	0.12	0.20	0.15	0.52	0.17	1.18	0.36
1.35-1.85	122	0.06	0.08	0.12	0.11	0.27	0.14	0.60	0.22	1.18	0.36
>1.85	555	0.02	0.03	0.07	0.06	0.24	0.10	0.64	0.13	1.22	0.27
**Smoking status**										
Yes	75	NA	NA	NA	NA	NA	NA	NA	NA	NA	NA
No	1,104	0.05	0.05	0.11	0.07	0.29	0.08	0.63	0.10	1.14	0.20

P10, P25, P50, P75, P90 represent the 10th, 25th, 50th, 75th and 90th percentile of intake. SE: Standard error. NA: not available (sample size too small to determine usual intake distribution)

Supplemental Table 2. Distribution of EPA and DHA intake (mg per day) from food alone and from food plus dietary supplements in childbearing-age women and pregnant women.

	Childbearing-age women – Food alone										
	**N**	**P10**	**SE**	**P25**	**SE**	**P50**	**SE**	**P75**	**SE**	**P90**	**SE**
**All**	10,285	23.23	2.18	36.59	2.42	58.74	2.38	92.17	3.12	136.37	6.81
**Age (years)**											
15-30	6,257	20.72	2.06	32.78	2.33	52.49	2.40	82.59	2.98	122.46	5.96
31-44	4,028	27.06	2.54	41.88	2.80	66.27	2.92	102.61	4.24	150.70	8.70
**Race**											
Non-Hispanic White	3,876	21.78	3.15	32.45	3.27	49.53	3.07	74.65	4.18	107.31	9.14
Non-Hispanic Black	2,477	27.60	3.58	46.60	4.53	79.31	5.74	130.32	8.25	201.65	14.72
Mexican Americans	2,250	30.41	5.10	45.72	5.37	69.06	5.21	100.88	6.51	140.31	12.29
**Education**											
Less than high school or equiv.	3,957	20.95	3.44	31.64	3.62	48.89	3.37	74.37	4.20	106.97	9.12
High school/equiv. & college/AA	4,680	22.27	3.48	35.83	3.80	58.03	3.51	91.58	4.12	135.84	9.49
College graduate & above	1,641	29.20	4.71	44.85	5.17	71.38	5.69	113.12	8.20	170.40	15.82
**Poverty income ratio**											
<1.35	3,934	22.23	2.76	35.33	2.85	54.84	2.90	81.84	4.18	115.64	8.33
1.35-1.85	1,100	12.45	2.66	23.06	3.44	43.37	4.47	79.59	7.11	132.91	15.57

>1.85	4,599	26.15	3.44	39.89	3.76	62.74	3.84	97.26	5.17	143.28	10.70
**Smoking status**											
Yes	1,580	15.77	3.27	25.39	4.02	42.16	4.47	67.65	4.88	102.00	8.00
No	8,442	25.97	2.65	40.18	2.83	63.61	2.75	98.82	3.78	145.26	8.25
	**Pregnant women – Food alone**										
	**N**	**P10**	**SE**	**P25**	**SE**	**P50**	**SE**	**P75**	**SE**	**P90**	**SE**
**All**	1,180	30.50	8.50	44.11	8.81	66.45	8.06	98.66	8.61	141.58	18.18
**Age (years)**											
15-30	864	29.12	8.43	42.08	8.89	63.64	8.39	94.56	9.18	134.90	17.71
31-44	316	33.31	9.47	47.71	10.04	71.10	10.15	105.20	13.12	152.24	24.48
**Race**											
Non-Hispanic White	494	27.32	12.76	36.07	12.07	48.55	9.75	62.96	7.15	75.89	17.55
Non-Hispanic Black	217	34.29	25.70	54.32	23.82	89.22	21.25	144.80	31.39	226.80	67.25
Mexican Americans	319	79.86	21.83	89.79	23.21	100.66	20.53	113.32	22.59	118.45	25.64
**Education**											
Less than high school or equiv.	367	63.44	13.61	84.42	20.56	112.06	23.23	123.42	26.28	126.16	27.30
High school/equiv. & college/AA	558	21.33	5.88	34.39	6.89	58.60	7.67	99.91	10.17	158.07	20.48
College graduate & above	255	21.75	10.43	32.85	11.22	51.74	11.92	80.79	18.60	120.56	40.31

**Poverty income ratio**										
<1.35	437	25.43	11.00	39.30	11.71	62.46	11.39	97.38	14.13	146.31	29.44
1.35-1.85	122	19.56	7.47	32.90	9.54	57.69	12.55	97.19	19.66	153.70	41.77
>1.85	555	37.92	19.93	52.86	19.20	75.44	13.71	106.16	14.49	142.58	35.45
**Smoking status**										
Yes	75	61.85	24.10	61.96	24.60	62.04	24.66	62.13	24.69	96.01	26.70
No	1,104	29.92	9.29	43.70	9.39	66.01	8.38	99.30	8.86	143.17	18.82

	Childbearing-age women – Food plus dietary supplements										
	**N**	**P10**	**SE**	**P25**	**SE**	**P50**	**SE**	**P75**	**SE**	**P90**	**SE**
**All**	10,285	23.36	2.17	37.04	2.39	60.16	2.33	97.13	3.43	154.78	8.87
**Age (years)**											
15-30	6,257	20.83	2.04	33.06	2.32	53.51	2.37	85.37	3.12	132.40	7.23
31-44	4,028	27.39	2.54	42.83	2.76	68.90	2.89	111.20	4.88	181.21	12.23
**Race**											
Non-Hispanic White	3,876	21.92	3.18	32.77	3.25	50.58	2.98	78.81	4.61	124.30	12.18
Non-Hispanic Black	2,477	27.92	3.60	47.06	4.54	80.03	5.74	132.35	8.47	207.52	15.28
Mexican Americans	2,250	30.56	5.09	46.17	5.37	70.01	5.30	103.86	6.72	149.33	13.70
**Education**											
Less than high school or equiv.	3,957	21.11	3.46	32.08	3.62	49.71	3.41	76.54	4.46	112.95	10.18
High school/equiv. & college/AA	4,680	22.22	3.51	36.06	3.79	59.02	3.59	95.30	4.62	149.30	11.70
College graduate & above	1,641	29.89	4.76	46.39	5.16	75.61	5.72	128.66	9.40	235.83	22.66
**Poverty income ratio**											
<1.35	3,934	22.47	2.76	35.68	2.83	55.83	2.91	84.98	4.47	124.95	9.94
1.35-1.85	1,100	12.52	2.65	23.48	3.46	44.50	4.68	83.05	8.26	148.80	19.46

>1.85	4,599	26.40	3.47	40.35	3.76	64.53	3.75	103.89	5.66	169.02	14.43
**Smoking status**											
Yes	1,580	15.89	3.28	25.85	4.00	42.94	4.49	70.39	5.43	111.29	10.63
No	8,442	26.07	2.69	40.62	2.83	65.13	2.70	104.46	4.10	166.49	10.80
	**Pregnant women – Food plus dietary supplements**										
	**N**	**P10**	**SE**	**P25**	**SE**	**P50**	**SE**	**P75**	**SE**	**P90**	**SE**
**All**	1,180	32.72	9.00	47.38	9.19	72.36	8.28	113.46	10.94	181.64	25.77
**Age (years)**											
15-30	864	30.86	8.76	44.30	9.05	66.10	8.48	99.87	9.72	149.01	21.80
31-44	316	38.82	10.79	56.29	11.48	87.39	13.32	145.33	22.82	244.25	44.20
**Race**											
Non-Hispanic White	494	25.83	12.64	35.31	11.13	49.66	7.81	72.86	10.56	127.15	28.80
Non-Hispanic Black	217	34.17	25.47	54.27	23.58	89.50	21.00	148.88	31.97	229.97	69.19
Mexican Americans	319	81.15	22.58	89.11	22.86	100.67	20.31	111.91	23.23	117.59	26.54
**Education**											
Less than high school or equiv.	367	64.12	14.26	87.55	22.09	114.59	24.17	124.62	26.93	127.78	28.13
High school/equiv. & college/AA	558	22.77	5.94	37.12	6.91	64.06	7.80	111.90	11.93	200.26	32.98
College graduate & above	255	24.07	11.63	37.83	12.78	61.95	15.06	111.52	31.66	214.38	58.26

Data source: National Health and Nutrition Examination Survey 2001-2014; usual intake estimated using the National Cancer Institute method

**Poverty income ratio**											
<1.35	437	25.50	11.14	39.72	11.89	64.18	11.33	102.10	14.63	160.01	34.40
1.35-1.85	122	20.46	7.97	34.61	10.02	59.65	13.00	103.31	24.27	178.95	70.03
>1.85	555	42.11	28.35	59.01	22.89	86.06	13.57	127.78	24.59	198.39	59.18
**Smoking status**											
Yes	75	61.33	23.93	61.97	24.61	62.45	24.87	63.04	25.09	96.01	26.61
No	1,104	32.20	9.88	47.24	9.97	72.42	8.75	115.45	11.33	192.05	26.90

P10, P25, P50, P75, P90 represent the 10th, 25th, 50th, 75th and 90th percentile of intake. SE: Standard error.
